# PET Imaging of Sphingosine-1-Phosphate Receptor 1 with [18F]TZ4877 in Nonhuman Primates

**DOI:** 10.21203/rs.3.rs-4350862/v1

**Published:** 2024-05-30

**Authors:** Jiwei Gu, Ming-Qiang Zheng, Daniel Holden, Krista Fowles, Lin Qiu, Zachary Felchner, Li Zhang, Jim Ropchan, Robert J Gropler, Richard E Carson, Zhude Tu, Yiyun Huang, Ansel T Hillmer

**Affiliations:** Yale University; Yale University; Yale University; Yale University; Washington University School of Medicine in Saint Louis: Washington University in St Louis School of Medicine; Yale University; Yale University; Yale University; Washington University School of Medicine in Saint Louis: Washington University in St Louis School of Medicine; Yale University; Washington University School of Medicine in Saint Louis: Washington University in St Louis School of Medicine; Yale University; Yale University

## Abstract

**Purpose:**

The sphingosine-1-phosphate receptor-1 (S1PR_1_) is involved in regulating responses to neuroimmune stimuli. There is a need for S1PR_1_-specific radioligands with clinically suitable brain pharmcokinetic properties to complement existing radiotracers. This work evaluated a promising S1PR_1_ radiotracer, [^18^F]TZ4877, in nonhuman primates.

**Procedures:**

[^18^F]TZ4877 was produced via nucleophilic substitution of tosylate precursor with K[^18^F]/F^−^ followed by deprotection. Brain PET imaging data were acquired with a Focus220 scanner in two *Macaca mulatta* (6, 13 years old) for 120–180 min following bolus injection of 118–163 MBq [^18^F]TZ4877, with arterial blood sampling and metabolite analysis to measure the parent input function and plasma free fraction (*f*_P_). Each animal was scanned at baseline, 15–18 min after 0.047–0.063 mg/kg of the S1PR_1_ inhibitor ponesimod, 33 min after 0.4–0.8 mg/kg of the S1PR_1_-specific compound TZ82112, and 167–195 min after 1 ng/kg of the immune stimulus endotoxin. Kinetic analysis with metabolite-corrected input function was performed to estimate the free fraction corrected total distribution volume (*V*_T_/*f*_P_). Whole-body dosimetry scans were acquired in 2 animals (1M, 1F) with a Biograph Vision PET/CT System, and absorbed radiation dose estimates were calculated with OLINDA.

**Results:**

[^18^F]TZ4877 exhibited fast kinetics that were described by the reversible 2-tissue compartment model. Baseline [^18^F]TZ4877 *f*_P_ was low (< 1%), and [^18^F]TZ4877 *V*_T_/*f*_P_ values were 233–866 mL/cm^3^. TZ82112 dose-dependently reduced [^18^F]TZ4877 *V*_T_/*f*_P_, while ponesimod and endotoxin exhibited negligible effects on *V*_T_/*f*_P_, possibly due to scan timing relative to dosing. Dosimetry studies identified the critical organs of gallbladder (0.42 (M) and 0.31 (F) mSv/MBq) for anesthetized nonhuman primate.

**Conclusions:**

[^18^F]TZ4877 exhibits reversible kinetic properties, but the low *f*_P_ value limits quantification with this radiotracer. S1PR_1_ is a compelling PET imaging target, and these data support pursuing alternative F-18 labeled radiotracers for potential future human studies.

## Introduction

Sphingosine-1-phosphate (S1P) is a sphingolipid that potently regulates brain immune responses, classically promoting cellular survival^1^. S1P affects neuroimmune function through activation with S1P receptors. The S1P system plays important roles in lymphocyte trafficking and vascular integrity^2^, and S1P modulators are approved for treating patients with multiple sclerosis and of interest for other conditions with altered brain immune function, such as amyotrophic lateral sclerosis, glioblastoma, and schizophrenia^3^. Of the five S1P receptor subtypes, S1PR_1_ is the most abundant in brain and is a G-protein coupled receptor. S1PR_1_ activation shifts brain immune state towards anti-inflammatory phenotypes, promoting cell survival, motility, and proliferation^4^. S1PR_1_ signaling also regulates key neuroimmune transcription factors such as nuclear factor-κB^5,6^, triggering cytokine and chemokine release and inhibiting histone deacetylases (HDAC1 and HDAC2)^7^. Indeed, inhibiting S1PR_1_ attenuates pro-inflammatory chemokine release^8^. Interestingly, pro-inflammatory conditions functionally upregulate S1PR_1_^9,10^, likely as a compensatory mechanism. Consequently, PET radiotracers that quantify S1PR_1_ *in vivo* would provide important data regarding S1PR_1_ target engagement, and potentially provide a biomarker for brain immune status.

**Figure 1:**
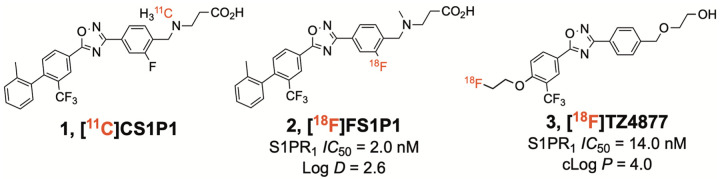
PET radiotracers for S1PR_1_

Several PET radiotracers specific for S1PR_1_ are reported. Importantly, S1PR_1_ is highly expressed in human brain, with estimated *B*_max_ values of 135±7 nM in post mortem frontal cortex^11^, which should be sufficiently high for quantitative PET imaging given a suitably specific radiotracer. The radiotracer [^11^C]CS1P1 (initially [^11^C]TZ3321; 1 in Fig. 1) exhibits elevated uptake in animal models of neuroinflammation^12–14^. These successes culminated in approval of [^11^C]CS1P1 for human imaging^15^, supporting its utility as a suitable radiotracer for imaging S1PR_1_ in human subjects.

Despite the current promise for [^11^C]CS1P1, there is important rationale for complementary PET radiotracers. [^11^C]CS1P1 does not exhibit fast kinetic properties^16^, while labeling with fluorine-18, which has a 109.8 min half-life, would allow for longer scan sessions that could improve quantification and facilitate human use at centers with no onsite cyclotron. To address this important need, several ^18^F-labeled compounds that target S1PR_1_ have been developed, including some based on [^11^C]CS1P1^16,17^ (see **2**, Fig. 1). Of these radiotracers, [^18^F]TZ4877 (**3**, Fig. 1) has an *IC*_50_ value of 14.01±0.05 nM for S1PR_1_ and selectivity over other S1PR subtypes (> 100-fold over S1PR_2 – 5_^18^), and in rat is well-described by the 2-tissue compartment model (2TCM) with reproducible estimates of volume of distribution (*V*_T_), with no evidence of radiolabeled metabolites penetrating the brain^17^. [^18^F]TZ4877 also exhibited elevated uptake in a mouse model of bacterial infection^19^ and rat model of experimental autoimmune encephalomyelitis (EAE)^17^. These data indicate that [^18^F]TZ4877 has potential for imaging S1PR_1_ in human subjects. To further examine this potential, we conducted studies with [^18^F]TZ4877 in nonhuman primates with the goals of characterizing its pharmacological and kinetic properties and estimating organ dosimetry.

## Methods

### Animals

The animals for this study were four *Maccaca mulatta* (1 female, 11 years old, 12.0 kg; 3 males, 6–13 years old, 10.3–18.0 kg). A total of 10 PET scans were conducted, including 8 brain-only acquisitions and 2 whole body acquisitions for dosimetry estimates. On the day of scans, subjects were initially sedated with combination of alphaxalone (1–2 mg/kg), midazolam (0.3 mg/kg) and dexmedetomidine (0.01 mg/kg) at least 2 h prior to radiotracer administration. Subjects were then maintained on oxygen and 1.5–3% isoflurane for the duration of scans. Vital signs, SpO2, and end tidal CO_2_ were continuously monitored and recorded, including respiration rate, blood pressure, heart rate, and temperature. All experiments followed institutional guidelines and were approved by the Yale University Institutional Animal Care and Use Committee.

### Radiochemistry

Radiosynthesis of [^18^F]TZ4877 was accomplished through nucleophilic radiofluorinaton starting with between the tosylate precursor to reach with [^18^F]KF/Kryptofix 222 in acetonitrile, followed by removal of the methoxymethyl (MOM) protecting group. [^18^F]Fluoride was produced via the ^18^O(p.n)^18^F nuclear reaction utilizing H_2_^18^O (Huayi Isotopes, Toronto, Canada) within a 16.5 MeV GE PETtrace cyclotron (Uppsala, Sweden). Following bombardment, the resultant activity was promptly transferred to a shielded hot cell and trapped on an anionic exchange resin cartridge (Chromafix PS-HCO_3_, Macherey-Nagel, Dueringen, Germany). The trapped [^18^F]F^−^ was subsequently eluted in a 2 mL borosilicate glass reaction vial using 1 mL solution comprising Kryptofix 222 (0.7 mL, 10 mg/mL in acetonitrile) and K_2_CO_3_ (0.3 mL, 2 mg/mL in deionized (D.I.) water. The solution was azeotropically dried for 5 min at 110°C under argon gas, followed by two rounds of drying with 1.0 mL MeCN under argon gas. To the dried [^18^F]F^−^ was then added a solution of the tosylate precursor (2 mg precursor in 0.4 anhydrous acetonitrile). The reaction mixture was stirred at 110°C for 15 min, followed by addition of 6 M HCl (150 μL) and heating at 110 °C for another 10 min. The reaction was cooled, quenched with 6 M NaOH (150 μL), and diluted with 1.2 mL of the HPLC mobile phase (47% acetonitrile in 0.1 M ammonium formate buffer, pH = 4.2). The mixture was then filtered through a Alumina N light Sep-Pak^®^ Cartridges (Pat No. WAT023561) and injected onto the semi-preparative HPLC (Waters Xbridge^®^BEH C18 column, 10 mm × 250 mm, 5 μm, UV = 254 nm, 4.0 mL/min). The HPLC fraction containing [^18^F]TZ4877 (retention time around 22–24 min) was collected, diluted with 50 mL of DI water, and then passed through a light C18 Sep-Pak^™^ cartridge (Pat No. WAT023501). The C18 cartridge was washed with 0.001 N HCl (10 mL) and dried with argon flow. The product was eluted off the C18 cartridge sequentially with 1 mL of USP ethanol and 3 mL of USP saline, and passed through a 0.22 μm membrane filter (GV, Millipore, Sigma) into a 10 mL dose vial pre-charged with 7 mL of USP saline and 8.4% NaHCO_3_ solution (20 μL). An aliquot of the final production solution was injected onto an analytic HPLC to determine radiochemical purity and molar activity. Tracer authentication was performed by co-injection with non-radiolabeled standard TZ4877 sample solution. The analytic HPLC conditions were: Phenomenex Luna C18(2) column, UV absorbance at 260 nm, with mobile phase of 55% CH_3_CN in 0.1 M ammonium formate with 5% AcOH (pH = 4.2) at a flow rate of 2.0 mL/min. The retention time of [^18^F]TZ4877 was 7.4 min.

### Brain PET Scanning Procedures

Brain-dedicated scans consisting of a baseline scan, preblocking scans with ponesimod and TZ82112, and an endotoxin challenge (see below) were acquired in each of two animals for a total of 10 brain studies. These data were acquired with a Focus 220 PET scanner (Simens/CTI, Knoxville, TN). A transmission scan was first acquired using a continuously rotating ^57^Co source for 9 min. [^18^F]TZ4877 was administered as a 118–163 MBq slow bolus injection over 3 min with a Harvard syringe pump (PHD 22/2000, Harvard Apparatus, Holliston, MA). PET data were then continuously acquired in list-mode for 120–180 min. An arterial line for blood sampling was inserted in a radial or femoral artery on the limb opposite the tracer administration line. Discrete arterial blood samples were acquired throughout PET scanning, with rapid (45 s) sampling immediately post-injection and gradually slowing to 30 min sampling at the end of scans.

### Arterial Input Function Measurement

Radioactivity assay of arterial blood samples was performed with a cross calibrated well-type gamma counter (Wizard 1480, Perkin Elmer, Waltham, MA). Whole blood samples were assayed and then centrifuged (2,930 *g* for 5 min). Plasma samples were then separated and assayed for radioactivity. Select plasma samples (drawn at 3, 8, 15, 30, 60, 90, 120, 180 min post-injection) were analyzed with HPLC to measure radioligand metabolism.

For metabolite analysis, plasma samples were mixed with urea to a final concentration of 8 M and filtered through 1.0 μm Whatman 13 mm CD/X filters (GE, Florham Park, NJ). Samples were then analyzed on an adapted column-switching HPLC system^20^. Upon injection, samples were first trapped on a C18 sorbent capture column (Strata-X, Phenomenex, Torrance CA) with a mobile phase of 1:99 (v:v) MeCN:H_2_O at 2 mL/min for 4 min. The capture column was then backflushed with a mobile phase of 45% acetonitrile and 55% 20 mM ammonium bicarbonate (v/v), and the eluent passed through a Phenomenex Gemini-NX analytical column (5 μm, 4.6 × 250 mm) at a flow rate of 1.65 mL/min. The eluent was collected with a fraction collector (CF-1 Fraction Collector, Spectrum Chromatography, Houston, TX) in discrete 2 min bins and counted in a gamma counter (Wizard 1480, Perkin Elmer, Waltham, MA). The fraction of unmetabolized parent was measured as the ratio of the eluted parent (retention time of ~12 min) to the total radioactivity collected. The time course of this parent fraction was fitted to an inverted gamma function and corrected for filtration efficiency. Finally, the input function was calculated as the product of the assayed radioactivity concentration in plasma and the unmetabolized parent fraction.

In addition, the free fraction (*f*_P_) was determined from plasma samples with ultrafiltration techniques. An arterial blood sample drawn prior to radiotracer injection (3.0 mL) was vigorously mixed with ~ 3 kBq of [^18^F]TZ4877. After partitioning the plasma from red blood cells via centrifugation, the plasma sample was extracted, loaded onto an ultrafiltration cartridge (Millipore Centrifree UF devices), and centrifuged at 1,228 *g* for 20 min. The free fraction was calculated as the ratio of radioactivity in the ultrafiltrate to the total radioactivity in the plasma sample. Measurements of *f*_P_ were performed in triplicate for each scan.

### Challenge Studies with [^18^F]TZ4877

To evaluate [^18^F]TZ4877 specific binding, blocking studies were acquired with each of ponesimod, and TZ8112, a compound with high affinity *(IC*_50_ value of 9 nM) and selectivity for S1PR_1_^21^. Ponesimod (0.047 or 0.063 mg/kg) was infused 15–18 min before [^18^F]TZ4877 injection, with PET image data acquired for 120 min. TZ8112 (0.4 or 0.8 mg/kg) was infused 33 min before [^18^F]TZ4877 injection, with PET image data acquired for 120 min.

To evaluate the effects of an acute immune stimulus on [^18^F]TZ4877 uptake, the classic acute immune stimulus lipopolysaccharide (LPS; NIH Clinical Center Reference *E. coli* serotype O:113) was used. A dose of 1 ng/kg, previously shown to trigger acute immune stimulus^22,23^, was administered 167–195 min before [^18^F]TZ4877 injection, with PET image data acquired for 180 min. Cytokine levels for TNF-α, IL-1β, IL-6, IL-8, IL-18, IL-22, IL-28A, and MCP-1 were measured in plasma samples taken 10 minutes prior to LPS administration and 90, 180, and 300 min after LPS injection. Cytokine levels were measured in triplicate using MILLIPLEX panel assay (MillporeSigma, Burlington, MA, USA).

### Anatomical MRI Acquisition

High-resolution T_1_-weighted images were acquired for image co-registration and region of interest (ROI) identification. MR data were acquired prior to PET image acquisition with a Siemens 3T Trio scanner, with an extremity head coil in the coronal direction and the following spin echo sequence (TE = 3.34 ms, TR = 2530 ms, flip angle = 7°, thickness = 0.50 mm, field-of-view = 140 mm, image matrix = 256 × 256 × 176, voxel size = 0.547 × 0.547 × 0.500 mm). Non-brain structures were removed with FMRIB’s Brain Extraction Tool (http://www.fmrib.ox.ac.uk/fsl/BET).

### Brain PET Data Processing

Raw list-mode PET data was histogrammed (frames of 6 × 0.5 min; 3 × 1 min; 2 × 2 min; and *N*×5 min to scan termination) and reconstructed with Fourier rebinning followed by 2D filtered back projection, using a Shepp filter and including corrections for scanner normalization, detector deadtime, randoms, scatter, and attenuation. This resulted in a reconstructed image resolution of ~ 3.2 mm. The PET images were then registered to MR image space with a 6-parameter rigid body registration^24^. The MR native space was then normalized using nonlinear affine registration to a high-resolution rhesus monkey atlas^25^ using BioImage Suite 3.01 (http://www.bioimagesuite.org/index.html). Time-activity curves were extracted by mapping atlas-defined regions to PET native space using the optimal transformation matrices calculated in the registration and normalization steps. ROIs extracted included caudate, cerebellum, frontal cortex, hippocampus, pons, putamen, temporal cortex, and thalamus.

### [^18^F]TZ4877 Kinetic Analysis

For all PET scans, the primary outcome measure was the total volume of distribution^26^, both uncorrected (*V*_T_) and corrected (*V*_T_/*f*_P_) by the plasma free fraction. Regional *V*_T_ values were estimated using both one-tissue (1TCM) and two-tissue (2TCM) compartment models (see^27^ for review). Model suitability was compared with the corrected Akaike Information Criterion (cAIC;^28^). Additionally, the multilinear analysis method (MA1;^29^) was assessed as a more stable, data-driven analysis method. To visualize [^18^F]TZ4877 *V*_T_ images, MA1 was also used to calculate V_T_ on the voxel level. Receptor occupancy *(Occ)* and nondisplaceable volume of distribution (*V*_ND_) was estimated with occupancy plots using the following Eq. 3^0^:

$$V_{\text{T}}/f_{\text{P}}(\text{baseline})-V_{\text{T}}/f_{\text{P}}(\text{block})=Occ(V_{\text{T}}/f_{\text{P}}(\text{baseline})-V_{\text{ND}}/f_{\text{P}})$$

### Radiation Dosimetry Study

The 2 whole body biodistribution scans were acquired in two animals (18.0 kg Male, 12.0 kg F). These data were acquired with a Biograph Vision PET/CT system (Siemens Medical Systems, Knoxville, TN) after i.v. injection of 94 MBq and 86 MBq [^18^F]TZ4877. Animals were imaged for approximately 3 hours in a sequence of 22 passes from top of the head to the mid-thigh. Images were reconstructed and visually inspected for organ activity concentrations exceeding background level. The organs included were heart, bladder, testes, kidney, liver, and gallbladder. Regions of interest were hand-delineated on these organs to compute mean time activity curves.

Within-pass decay correction was removed to reflect the actual activity in each organ, and the tail portions of each curve beyond the end of the scan were extrapolated assuming only physical decay of the radiotracer. The cumulative activity (Bq·h/cm^3^) was computed by integrating these data. These values were multiplied by the organ volumes of standard 60 kg adult female and 73 kg adult male reference mathematical phantoms^31^ and normalized to injected activity to obtain organ residence times. Residence times were then entered into OLINDA/EXM 2.0 software to compute absorbed doses in all organs^32^, which were computed without voiding.

## Results

### Radiochemistry

The synthesis of [^18^F]TZ4877 was accomplished with high chemical and radiochemical purity (> 99%), good radiochemical yield (5.46 ± 0.68%, n = 9), and high molar activity (284 ± 136 GBq/μmol, n = 9, decay corrected to the end of synthesis, EOS).

### [^18^F]TZ4877 in Arterial Plasma

**Figure 3:**
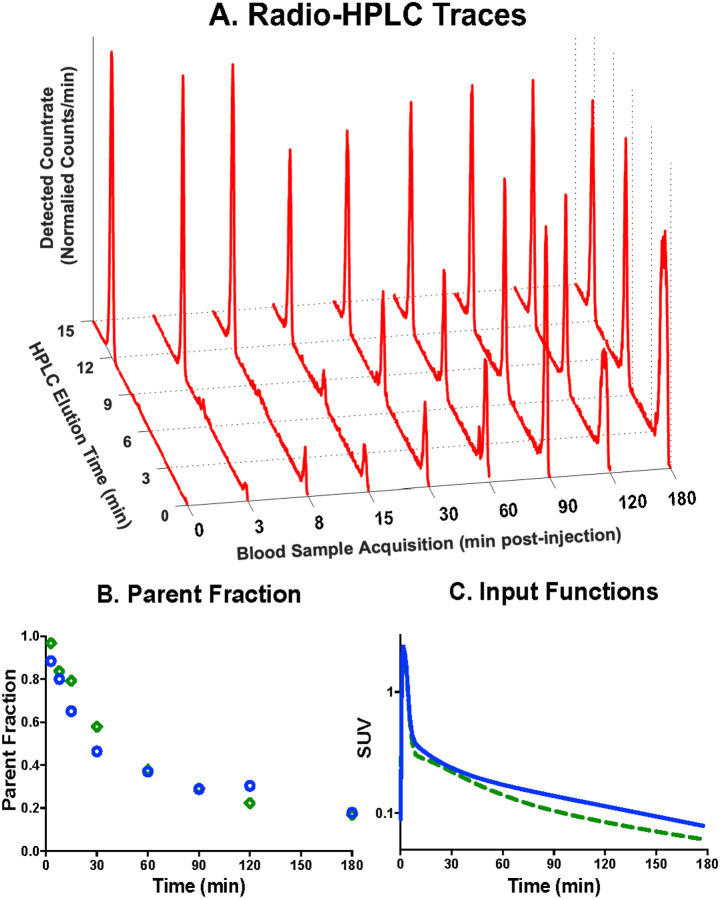
[^18^F]TZ4877 in the Arterial Plasma. **A** Shows the radioactive HPLC chromatogram for a baseline scan, with retention time of parent [^18^F]TZ4877 at ~12 min and elution of metabolites at ~7 min and ~1 min. **B** shows the measured [^18^F]TZ4877 parent fractions at baseline. **C** shows the [^18^F]TZ4877 input functions at baseline in units of SUV (activity normalized by injected dose and subject weight). Different symbols/colors distinguish the two study animals.

Analysis of radiolabeled [^18^F]TZ4877 metabolites in arterial plasma was performed with HPLC. Radiometabolites appeared to be more polar than [^18^F]TZ4877 (see Fig. 3A). [^18^F]TZ4877 parent fraction was consistent across the two subjects, at 37–38% and 17–18% at 60 min and 180 min post-injection, respectively (see Fig. 3B). The calculated parent input functions are shown in Fig. 3C. The free fraction, *f*_P_, of [^18^F]TZ4877 at baseline was low (0.87% and 0.56%).

### [^18^F]TZ4877 Brain Tissue Kinetics

**Figure 4:**
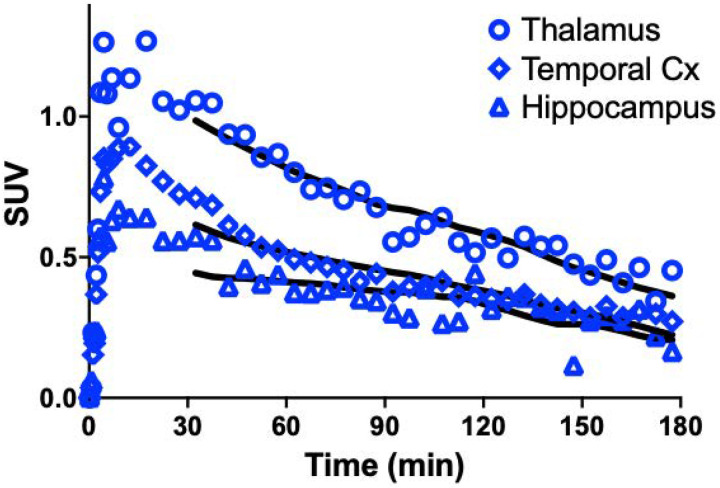
[^18^F]TZ4877 Tissue Time Activity Curves. Values are expressed in SUV (radioactivity concentration/i.d. × weight × 1,000). Open symbols are the measured concentrations, while solid lines show the fit with MA1 (t*=30 min).

**Figure 5:**
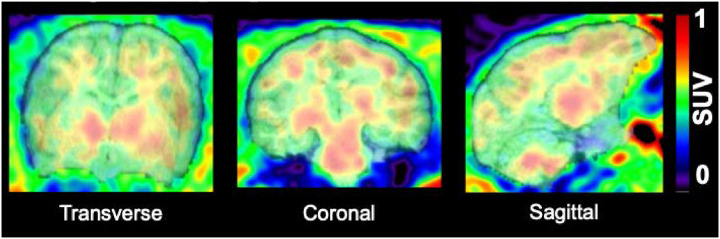
[^18^F]TZ4877 Uptake in the Rhesus Monkey, shown in SUV (radioactivity concentration/i.d. × weight × 1,000) at 60–90 min post-injection.

Uptake of [^18^F]TZ4877 in the brain occurred rapidly, peaking at SUVs of 0.6–1.3 within 20 min post-injection and followed by clearance from the brain (**see** Fig. 4). Higher uptake was observed in thalamus and putamen, moderate uptake was observed in neocortical brain regions, and low uptake was observed in hippocampus and cerebellum (see Fig. 5).

Compartment modeling of [^18^F]TZ4877 TACs with the 1TCM produced visually poor curve fits, while use of the 2TCM (reversible uptake) produced visually good curve fits but did exhibited high (> 100%) standard error in estimates of [^18^F]TZ4877 *V*_T_. Therefore, estimation of [^18^F]TZ4877 *V*_T_ was performed with the MA1 method using t*=30 min, which yielded regional baseline *V*_T_ values of 2.0–4.8 mL/cm^3^ (see Table 1). For regions with stable 2TCM estimates, *K*_1_ values ranged from 0.07 to 0.15 mL·cm^−3^·min^−1^.

### Blocking Studies of [^18^F]TZ4877

Administration of the S1PR_1_ inhibitor ponesimod prior to [^18^F]TZ4877 PET scans unexpectedly increased [^18^F]TZ4877 *V*_t_ by 22±13% and 15±9%. [^18^F]TZ4877 *f*_P_ was unaffected by ponesimod. Administration of the S1PR_1_-selective compound TZ82112 increased [^18^F]TZ4877 *V*_T_ by 20±9% and 10±5%, however, [^18^F]TZ4877 *f*_P_ also increased in a dose-dependent manner such that using [^18^F]TZ4877 *V*_T_//*f*_P_ as an outcome measure yielded a dose dependent reduction. Resultant occupancy plots exhibited linearity and yielded estimated occupancies of 49% (0.4 mg/kg dose) and 73% (0.8 mg/kg dose), with *V*_ND_/*f*_P_ values of 41–95 mL/cm^3^ (see **Supplementary Fig. 1**). These results should be used with caution due to the very low *f*_P_ values.

### LPS Challenge Studies with [^18^F]TZ4877

Administration of the classic immune stimulus LPS increased [^18^F]TZ4877 *V*_T_ in both animals by 26±11% and 7±3%. However, *f*_P_ appeared relatively unaffected, and changes in [^18^F]TZ4877 *V*_T_/*f*_P_ were variable (32±10% and - 7±4%). MCP-1 concentrations increased in both animals (8.6–9.7 fold) at the time of [^18^F]TZ4877 scan start (see **Supplementary Table 1**). in addition, both TNF-α and IL-6 increased relative to baseline for M1, while M2 results were difficult to interpret as baseline cytokine levels were near or below detection thresholds.

### Radiation Dosimetry Biodistribution Estimates

Whole-body distribution studies were performed in two rhesus macaques and revealed highest uptake of [^18^F]TZ4877 in the gallbladder and liver. Organ residence times are presented in **Supplementary Table 2**, with the liver yielding the highest mean integrated time-activity curve (0.91–1.0 h; 35–38% of injected dose). Absorbed doses estimated for the male and female phantom are presented in **Supplementary Table 3**. The organs receiving the largest doses were the gallbladder (0.31 mSv/MBq and 0.17 mSv/MBq for F and M, respectively) and the liver (0.16 mSv/MBq and 0.12 mSv/MBq for F and M, respectively). Based on the gallbladder as the critical organ, the maximum permissible single-study dosage of [^18^F]TZ4877 to remain below the 21 CFR 361.1 dose limit would be 160 MBq (4.31 mCi) for female and 118 MBq (3.20 mCi) for male. The estimated effective dose (ED) is 0.023 mSv/MBq for female and 0.020 mSv/MBq for male. Based on these estimates and assuming direct translation of anesthetized rhesus macaque dosimetry to humans, a single study administering a conservative 115 MBq [^18^F]TZ4877 would result in an effective dose of 2.6 mSv, with a gallbladder dose of 47.8 mSv.

## Discussion

PET radiotracers targeting S1PR_1_ could provide important tools to evaluate target engagement with S1PR_1_ modulators and evaluate this receptor in conditions featuring brain immune changes. The radiotracer [^11^C]CS1P1 is approved for human use^15^, and an F-18 labeled version has been characterized in nonhuman primates^16^. These radiotracers exhibit high brain uptake with no radiolabeled metabolites, however, they also exhibit relatively slower tracer kinetics in brain and do not achieve transient equilibrium within two-hours post-injection^16^. These properties motivate the development of complementary radiotracers with faster kinetics to facilitate improved quantification. [^18^F]TZ4877 exhibits rapid brain uptake followed by washout, consistent with fast radiotracer kinetics. Indeed, TZ4877 is roughly an order of magnitude less potent than CS1P1 *(IC*_50_ = 14.0 nM vs 2.0 nM^18^) and exhibits a much faster reduction in parent fraction, which are both characteristics that likely contribute to the faster kinetic properties.

In addition to its fast kinetics, binding of [^18^F]TZ4877 was displaced by the S1PR_1_-specific ligand TZ82112. However, quantification of [^18^F]TZ4877 blockade required incorporation of the plasma free fraction (*f*_P_) term, making [^18^F]TZ4877 *V*_T_/*f*_P_ the primary outcome measure. [^18^F]TZ4877 *f*_P_ is very low (< 1%) at baseline conditions, which makes it challenging to measure [^18^F]TZ4877 *V*_T_/*f*_P_ with good precision. Alternative radiotracers with higher *f*_P_ values and/or *V*_T_ as the outcome measure would therefore be preferable for quantification of S1PR_1_ in human subjects. Interestingly, the S1PR_1_ inhibitor ponesimod did not effectively compete with [^18^F]TZ4877 for binding to S1PR_1_ when infused ~ 15 min prior to injection. This may be due to its poor brain penetration^33^, was ponesimod has an estimated logP of 4–4.5, or insufficient dosing, as administration of the targeted dose (0.1 mg/kg) was halted due to rapid drop in animal heart rate. Ponesimod was nonetheless selected for blocking because it presents a favorable safety profile compared with other S1PR_1_ inhibitors^34^ and was previously shown to displace other S1PR_1_ radiotracers^35^. Thus, performing PET imaging later after ponesimod dosing, or using a compound with better brain penetration (e.g., siponimod^36^, fingolimod^37^), could provide improved evaluation of S1PR_1_ blocking in future evaluation of S1PR_1_ radiotracers, but must be balanced with animal safety concerns.

Administration of the classic immune stimulus endotoxin did not produce noticeable effects on [^18^F]TZ4877 *V*_T_/*f*_P_, although [^18^F]TZ4877 *V*_T_ did increase (with variable magnitude) in both animals. Since [^18^F]TZ4877 *V*_T_/*f*_P_ is required to observe displacement, we conclude that this radiotracer was not sensitive to endotoxin effects at this dose (1 ng/kg) and timing (~ 3 hours pre-injection). This dosing plan elicits robust increases in levels of the 18-kDa translocator protein (TSPO), as measured with [^11^C]PBR28 *V*_T_, in both rhesus monkey^22^ and humans^38^. The presented data indicate a robust MCP-1 response to 1 ng/kg endotoxin after 3 h, although other classic cytokines (e.g., IL-8) were more variable than previously reported^23^. Indeed, Previously published *in vitro* data demonstrate increased S1PR_1_ radiotracer binding in brain^39^ and liver^40^ roughly 24 hours after 15 mg/kg LPS in mice, and S1PR_1_ reporter mice exhibit enhanced activation of S1PR_1_ after LPS stimulation^41^. Notably, LPS effects of dose and timing vary significantly across species and biological target^42^, thus it is possible that the timing of maximal S1PR_1_ effect to this LPS dose could be optimized for rhesus monkey.

Dosimetry studies identified ED estimates of 0.023 mSv/MBq for female and 0.020 mSv/MBq for male, with the gallbladder as the critical organ. Based on the estimated gallbladder doses, the maximum single-study dose would be 118 MBq to remain compliant with a 50 mSv organ maximum. This dose would allow for a maximum of 3 studies of 118 MBq [^18^F]TZ4877 each to not exceed an annual organ dose of 150 mSv. However, higher radiotracer residence times in digestion-related organs, including gallbladder, for anesthetized nonhuman primates relative to humans have been reported previously, likely due to decreased gastrointestinal motility from anesthesia^43^. Indeed, human dosimetry studies with [^11^C]CS1P1 reported the liver as the critical organ^15^, and we speculate the liver may be the critical organ for [^18^F]TZ4877 in non-anesthetized humans.

To summarize, we present an evaluation of [^18^F]TZ4877 imaging properties in non-human primates. This radiotracer exhibits fast and reversible kinetic properties, but requires measurement of *f*_P_ for full quantification. Additionally, dosimetry estimates from anesthetized nonhuman primates would limit studies to injections of 118 MBq [^18^F]TZ4877, although anesthesia and translational differences may result in a different profile for non-anesthetized humans. Taken together, although [^18^F]TZ4877 provides a useful imaging tool for quantification of S1PR_1_ in preclinical models, alternative F-18 radiotracers^16,17^ may provide more suitable imaging properties and are of high interest for imaging S1PR_1_ in potential future human studies.

## Figures and Tables

**Figure 2:**
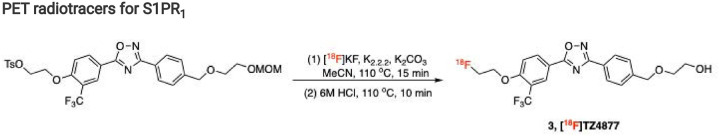
Radiosynthesis of the S1PR_1_ specific radiotracer [^18^F]TZ4877

**Table 1:** [^18^F]TZ4877 total distribution volume ([^18^F]TZ4877 *V*_T_, mL/cm^3^) estimates, with and without correction for the free fraction, are shown based on multilinear analysis (MA1, t*=30min) for both animals under baseline, blocking (ponesimod and TZ82112), and LPS challenge conditions.

		Baseline		Ponesimod		Cold TZ82112		LPS (1 ng/kg)	
		M1	M2	M1 (0.047 mg/kg)	M2 (0.063 mg/kg)	M1 (0.4 mg/kg)	M2 (0.8 mg/kg)	M1	M2
*V*_T_ (mL/cm^3^)	Caudate	2.6	3.8	3.7	5.3	3.1	4.2	3.5	4.3
	Cerebellum	2.3	3.3	3.3	3.7	3.1	4.0	3.3	3.5
	Frontal Cx.	4.0	3.9	3.9	5.2	4.3	4.1	4.2	4.4
	Hippocampus	2.0	2.9	3.6	3.0	3.0	3.2	3.4	3.0
	Pons	2.2	3.4	2.8	3.6	2.9	3.9	3.6	3.5
	Putamen	3.6	4.8	4.4	5.5	5.3	5.0	5.3	5.0
	Temporal Cx.	3.1	4.3	3.6	5.4	3.4	4.4	3.9	4.5
	Thalamus	3.7	4.7	4.5	5.5	4.2	5.4	4.9	5.0
	*f*_P_ (%)	0.87	0.56	0.64	0.61	1.67	1.96	0.81	0.64
									
*V*_T_/*f*_P_ (mL/cm^3^)	Caudate	301	682	582	868	187	217	429	671
	Cerebellum	269	591	520	600	187	203	407	548
	Frontal Cx.	454	690	611	846	258	211	521	685
	Hippocampus	233	516	570	484	178	161	421	470
	Pons	253	604	443	589	172	201	442	553
	Putamen	417	866	687	897	317	256	650	779
	Temporal Cx.	355	759	559	881	205	223	480	709
	Thalamus	429	833	705	897	254	277	607	784

## Data Availability

The authors declare that the data supporting the findings of this study are available within the paper and its Supplementary Information files. Should any raw data files be needed in another format they are available upon request.
